# Comparisons of Activity Budgets, Interactions, and Social Structures in Captive and Wild Chimpanzees (*Pan troglodytes*)

**DOI:** 10.3390/ani10061063

**Published:** 2020-06-19

**Authors:** Nodoka Inoue, Masaki Shimada

**Affiliations:** Department of Animal Sciences, Teikyo University of Science, 2525 Yatsusawa, Uenohara, Yamanashi Prefecture 409-0193, Japan; d-magic.mn-30@i.softbank.jp

**Keywords:** chimpanzee, Tama Zoological Park, Mahale Mountains National Park, social network, social grooming, environmental enrichment

## Abstract

**Simple Summary:**

For chimpanzees in zoos, the key aim of environmental enrichment is to enable them to exhibit behaviors, interactions, and societies similar to chimpanzees in the wild. A comparison of observational data, showed that the proportion of their time spent on collecting foraging was significantly lower in captive chimpanzees (located in Tama) than in wild chimpanzees (located in Mahale), but no significant difference was found between the two groups in terms of the proportion of their total time spent collecting, extractive, and hunting foraging. The percentage of time spent performing mutual grooming was much higher in Tama than in Mahale. Males, but not females, in Mahale formed a core social group, but no sex-specific differences were found in Tama. The multiple artificial feeders allowed chimpanzees in Tama to spend more time on extractive foraging to achieve a similar proportion of time on foraging as compared with that of the wild chimpanzees. The environmental enrichment for chimpanzees in Tama can be considered to be successful.

**Abstract:**

Chimpanzees in zoos with sufficient and appropriate environmental enrichment devices are expected to exhibit behaviors, interactions, and societies similar to those in the wild. In this study, we compared the activity budgets of each observed behavior, characteristics of social grooming, and social networks of captive chimpanzees at Tama Zoological Park (Tama) with those of wild chimpanzees at Mahale Mountains National Park, Tanzania (Mahale), and tested our predictions. We surveyed 16 chimpanzees in both Tama and Mahale and recorded the behaviors and individuals in proximity of each focal individual and social grooming the focal individuals participated in. The proportion of time spent collecting foraging was significantly lower in Tama than in Mahale. Additionally, the percentage of mutual grooming was much higher in Tama than in Mahale. All focal individuals in Mahale performed mutual grooming interactions, including grooming handclasp (GHC) but this was not observed in Tama. The result of a high rate of mutual grooming in chimpanzees in Tama without GHC and the finding that individuals forming the core of their social network are sex independent suggest that chimpanzees placed in an appropriate environmental enrichment have idiosyncratic grooming or social features, even in captivity.

## 1. Introduction

### 1.1. Environmental Enrichment for Captive Chimpanzees

Environmental enrichment can be defined as any proactive effort to improve the living environment of captive animals based on knowledge of their behavior and ecology in the wild [1,2]. One of the ultimate goals of environmental enrichment in captive animals is to bring their behavioral repertoire and daily behavioral time budgets closer to those of their wild counterparts [3]. Environmental enrichment for captive primates should encompass all five of the following categories: social, physical, nutritional, occupational, and sensory [1,4]. For example, devising enrichment in the form of an improved feeding method or using more appropriate feeders can be thought of as nutritional, occupational, and sensory enrichment [3,5,6]. Likewise, the installation and introduction of three-dimensional structures and playground equipment can be considered to be occupational and physical enrichment [7]. In addition, rearing multiple individuals in a group can be thought of as social enrichment [4,6,8].

Zoos in Japan have been working to improve their environmental enrichment for captive chimpanzees. For example, Tama Zoological Park, Tokyo (Tama) provides many forms of enrichment for their captive chimpanzees based on the criteria mentioned above. The introduction of several types of enrichment items in Tama allows chimpanzees to spend more time foraging and fulfills the aims of physical, occupational, and sensory enrichment [5,9,10]. Foraging was subdivided into the following three categories: collecting, extractive, and hunting foraging [11]. Collecting foraging is the behavior of directly eating food resources, such as fruits and leaves, collected from the environment. Extractive foraging is a series of behaviors, such as preparing tools to extract, remove, process, and eat hidden food resources, such as roots, the inside of hard nuts or seeds [12], and invertebrate animals such as carpenter ants that live inside trees [13]. Hunting foraging is a series of behaviors such as chasing and catching mobile vertebrate animals, and sharing or eating their meats [14,15]. For example, the so-called “artificial anthill” in Tama is a feeder with juice inside, which can be acquired as each individual goes through different steps, such as obtaining a stick to insert by procuring a tree branch, inserting and pulling the stick, and licking juice from the stick [16]. Artificial anthills have succeeded in causing chimpanzees to exhibit extractive foraging, which is similar to the ant-fishing behavior observed in some groups of wild chimpanzees, such as the ones in Mahale Mountains National Park, Tanzania (Mahale). Chimpanzees in Mahale create probes from plant materials, insert them into the entrance of the nest of wood-boring carpenter ants (*Camponotus* spp.), then, withdraw the probes and eat the ants [13,17,18].

Few studies have clarified the effects of environmental enrichment. Previous studies on chimpanzees in Tama have been conducted on the process of acquiring skills for nut cracking and the parenting behavior of mothers [19,20]. However, there has been no quantitative comparative study conducted on the activity budgets, social grooming, or social structure of captive Tama chimpanzees and those of wild groups.

### 1.2. Purpose of Research and Predictions

The purpose of this study was to test the hypothesis that captive chimpanzees living under conditions with appropriate environmental enrichment would behave, interact, and form social structures in a similar manner to wild chimpanzees, and to determine the effect and function of environmental enrichment on captive chimpanzees. To test this hypothesis, we compared the activity budgets, grooming interaction features, and social network of captive chimpanzees at Tama with those of wild chimpanzees in Mahale and tested the following predictions.

In Tama, the proportion of time spent on collecting foraging is expected to be lower than that of chimpanzees in Mahale, since captive chimpanzees eat scheduled baits or food items given by zookeepers at regular intervals while wild chimpanzees forage for food resources in the field themselves. In contrast, because of the zoo environment in Tama, hunting is impossible, but chimpanzees are still expected to spend much of their time on extractive foraging because of the installation of multiple artificial feeders [16]. Therefore, the percentage of time spent foraging, that is, the total time for collecting, extractive, and hunting foraging, is expected to be comparable to that of the chimpanzees in Mahale. Because of the comparatively smaller size of the outside enclosure (0.23 ha), the percentage of time spent by Tama chimpanzees on traveling is expected to be lower than that of the Mahale chimpanzees.

Mutual grooming is a form of social grooming in which the amounts of grooming individuals give and receive from a partner are inevitably the same [21,22]. When two individuals exchange one-directional grooming, the amount of grooming is mutually beneficial only if the two individuals meet in the long term, but when the chance of meeting in the future is uncertain, it is more rational to exchange mutual grooming and receive the same amount of grooming from the partner at the same time [23,24]. Therefore, chimpanzees in Tama, where each individual can be in close proximity to all other individuals, are predicted to have a lower proportion of mutual grooming than chimpanzees in Mahale, where it is always possible to be away from each other at great physical distances for a long period of time [25,26].

To avoid lethal aggressive interactions between captive males (e.g., [27]), the zookeepers in Tama decide, on a daily basis, which combination of individuals to keep in the outside enclosure, out of the animals kept in Tama. Therefore, in Tama, there is no need for males to form alliances and compete with other males for a competitive advantage [28,29]. In addition, there are no externally hostile neighboring groups, and the need for males to form strong social bonds with each other or alliances is also lower than in Mahale [30]. Because proximity is known to be an indicator of an affiliative relationship between individuals [31], male–male proximity is predicted to be higher than female–female proximity or male–female proximity in Mahale, but this is not the case in Tama [32,33,34]. Finally, chimpanzees in Mahale, similar to other wild groups, are predicted to form a male-dominated society. In other words, the males take central positions and form the core of the social network of the members in Mahale, but no such tendency is predicted in Tama [35].

## 2. Methods and Materials

### 2.1. Subjects and Observational Methods

The first author (NI) conducted observations on Tama chimpanzees from April to October 2019, with 42 observation days in total. In Tama, there were 19 captive individuals in total during the study period. Out of the 19 individuals, 5 males (1 adult, 3 young, and 1 juvenile) and 11 females (10 adults and 1 young) that were routinely released into outdoor enclosure were selected as the focal individuals in our study (Table 1). The zookeepers decided which individuals to exhibit in the outdoor enclosure each day based on the estrus cycle of each female chimpanzee, while the other individuals were kept in private enclosures in the back yard. Approximately 10 out of the 16 focal individuals were exhibited daily in the outdoor enclosure in areas measuring 0.23 ha. There are two 15 m tall steel towers on the premises, and three-dimensional structures such as wooden towers [16,19]. The presence of a central hill in the outside enclosure allowed each individual to create blind spots and avoid excessive gazing from visitors in the zoo. In the outdoor enclosures, there are many enrichment items associated with foraging, such as so-called “bamboo tube feeders”, “braided fire hoses”, “UFO catchers”, and “artificial anthills,” all of which have mechanisms that allow the chimpanzees to exercise complex movements and spend a long time obtaining the food resources found inside [16,19,20].

Mahale is located on the eastern shore of Lake Tanganyika, at the western end of Tanzania in Africa. The entire area of Mahale is approximately 1600 km^2^, where more than 10 independent groups are distributed. One of groups, the M group, has an area of approximately 2700 ha [36]. The second author (MS) conducted observations on Mahale chimpanzees from October 2001 to February 2002, with 69 observation days in total. In the M group in Mahale, there were 55 individuals in total during the study period. Eight males (7 adults and 1 young) and eight adult females were selected as the focal individuals for the wild group out of the 55 chimpanzees (Table 1).

In both groups, we conducted observations using the focal sampling method [37]. The focal animal was changed approximately every 5 h in Tama and every 2 h in Mahale. Before we began observing, focal animals were selected so that there was as little sampling bias as possible among the three time periods per day (until 12 am, 1 pm to 2 pm, after 2 pm) and total observation days in each group. When observing animals in the field or outside enclosure, the focal individual was not always within the view of the observer. Therefore, for the recording of behaviors and proximate individuals, it was reasonable to use the instantaneous sampling method or one-zero sampling method with appropriate sampling intervals according to the observation conditions, rather than the continuous sampling method [37]. In Tama, during the daytime when chimpanzees were in the outside enclosure, the behaviors of the focal animals and the individuals approaching within 3 m of them were recorded using the instantaneous sampling method at 3 min intervals. In Mahale, the behaviors of the focal animals were recorded using the instantaneous sampling method at 1 min intervals, and the individuals approaching within 10 m of these focal animals were recorded using the one-zero sampling method at 5 min intervals. In both groups, the activities of chimpanzees were recorded according to the following categories: foraging, traveling, resting, social grooming, and others (all the remaining activities that cannot be classified into these four categories, according to the ethogram of wild chimpanzees) [38]. The feeder-using behavior in Tama (processing a probe or stick, inserting it into artificial anthill, and licking the fruit juice) was defined as extractive foraging, as was the ant-fishing process in Mahale [13]. “Others” included activities such as drinking water, solitary play, social play, vocalization, or aggressive behaviors. Abnormal behaviors, such as coprophagy, hair plucking, self-injurious behavior, or pacing, were recorded if observed [5,39,40].

In both groups, we used all occurrence sampling methods to record sequences of social grooming interactions, including focal individuals, in seconds. A grooming clique was defined as a configuration of directly connected individuals through grooming interactions at a given moment [22]. We used a modified version of this definition to record only observed social grooming, including focal individuals that directly groomed the other individual or were directly groomed by the other individuals as a sequential chain of grooming cliques. The types of grooming cliques were coded by a three-digit number, according to Nakamura [41]. The leading number indicated whether the focal individual was mutually groomed or not, and the number in the middle indicated whether the focal individual was grooming the partner or not; we gave a value of 1 if it was, and 0 if it was not. The last number indicated the number of individuals grooming the focal individual.

Furthermore, the grooming handclasp (GHC) is known as an interactive pattern of mutual grooming in which both individuals groom around the underarm of another individual with either one clasping the other’s corresponding wrist, arm, or hand, by crossing wrists or clasping each other’s hands on their heads [42,43,44]. In each group, we checked whether or not each focal individual engaged in mutual grooming, including GHC.

### 2.2. Variables and Data Analysis

The activity budget of each behavior category in the total observation time of each individual was calculated for each group.

Using the data of individuals in close proximity to the focal individual, a proximity index (*PI_AB_*) between individuals *A* and *B* was applied to the following Equation (1) and calculated as a simple proximity ratio [35]:

(1) $$PI_{AB}=\frac{T_{A\left(B\right)}+T_{B\left(A\right)}}{T_{A}+T_{B}}$$

where *T_A_* represents the total observation time of focal individual *A*, *T_A_*_(*B*)_ represents the total time the focal individual *A* is close to *B*, while *A* is observed. 

Adjacency matrices for both groups were created using *PI*s between 16 individuals. When drawing and analyzing social networks, once all the proximity relationships are left in and the same group is observed over a long period of time, as in this study, all individuals are connected to each other and miss a meaningful social structure. Thus, filtering was performed so that only *PI*s with values equal to or higher than the third quartile remained [37]. Non-directed networks were created using the relationships left by the filtering, and they were regarded as representative social networks of chimpanzees in Tama and Mahale [35,45]. Social network analysis was conducted for these networks, and the eigenvector centralities of each individual were calculated in both groups.

The amounts of mutual grooming and total social grooming for each individual were calculated in seconds, and the mutual grooming ratio was defined as the value of the former divided by the latter. Data for individuals that were rarely observed participating in any social grooming, as well as those for a juvenile individual (fu), were excluded from the analysis.

### 2.3. Statistical Analyses

The activity budget of each behavioral category and the mutual grooming ratio of each individual were compared between groups (Tama/Mahale) using a nonparametric test (Mann–Whitney’s *U* test). One activity budget and one mutual grooming ratio per individual were included in each analysis.

Since the data used to derive the *PI*s were different between Tama and Mahale, the *PI*s could not be compared directly between groups. Therefore, the *PI*s of each individual were compared with the sex combinations (male–male/female–female/male–female) using a nonparametric test (Kruskal–Wallis test). When the result was significant, multiple comparisons by Holm’s method were performed.

For the comparison of eigenvector centralities between males and females in each group, we used randomization test [46]. Firstly, we performed Welch’s two sample *t*-test for observed eigenvector centralities between males and females to obtain observed *t*-values. Then, we performed 10,000 resamples of the eigenvector centrality of each sex, calculated the resampled t-values for each by the Welch’s method, and examined the p-value of the obtained *t*-values that were smaller than the observed *t*-value, using the distribution of resampled t-values.

Data were analyzed using the statistical freeware HAD [47], UCINET6.0, NetDraw 2.166 [48], and in R (R Core Team, version 3.4.4). The significance level was set at α = 0.05.

### 2.4. Ethical Approval

To conduct field research on wild chimpanzees in Mahale, M.S. complied with protocols approved by the Tanzania Wildlife Research Institute (TAWIRI) and adhered to the legal requirements of the government of Tanzania. Our research on captive chimpanzees in Tama was approved by the Teikyo University of Science Animal Committee (no. 19C027) and permitted by Tama Zoological Park. Our research adhered to the American Society of Primatologists (ASP) Principles for the Ethical Treatment of Non-Human Primates.

## 3. Results

### 3.1. Activity Budget

**Figure 1:**
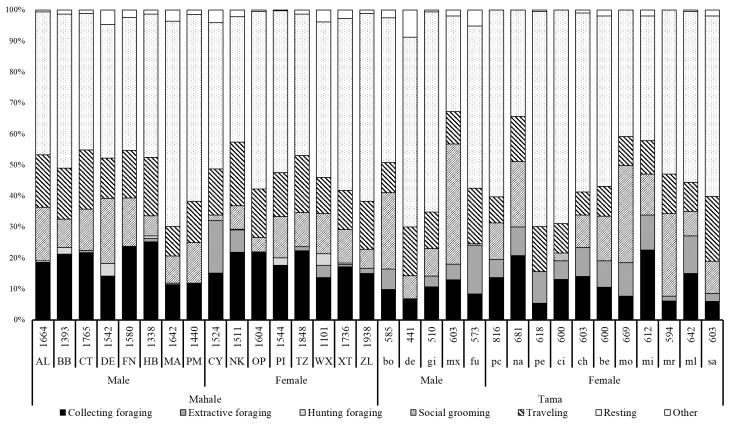
Percentage of time in each behavioral category for each individual in both groups. The numbers above the abbreviations of the focal individuals’ names indicate the total number of minutes that each individual was observed.

The mean +SD observation time per subject was 1573.1 ± 197.2 min (n = 16) in Mahale and 609.4 ± 76.3 min (n = 16) in Tama (Table S1). All chimpanzees in both groups were in good health during the observation period, and no abnormal behavior was observed. The activity budget of each behavioral category for each focal individual in both groups is shown in Figure 1.

The activity budgets of collecting foraging for each individual in Tama (11.4 ± 4.9%) were found to be significantly lower than those in Mahale (18.2 ± 4.2%) (Mann–Whitney’s *U* test, *U* = 37.0, df = 1, *p* < 0.001). However, the activity budgets of all the foraging types that combined collecting, extractive, and hunting foraging were found to be not significantly different between the two groups (Tama 18.8 ± 7.4% and Mahale: 21.3 ± 5.3%, Mann–Whitney’s *U* test, *U* = 98.0, df = 1, *p* = 0.266).

The activity budgets of traveling for each individual in Mahale (Mahale 15.4 ± 2.9%) were found to be higher than those in Tama (12.0 ± 3.6%) (Mann–Whitney’s *U* test, *U* = 57.0, df = 1, *p* = 0.008).

### 3.2. Social Grooming and Mutual Grooming

**Figure 2:**
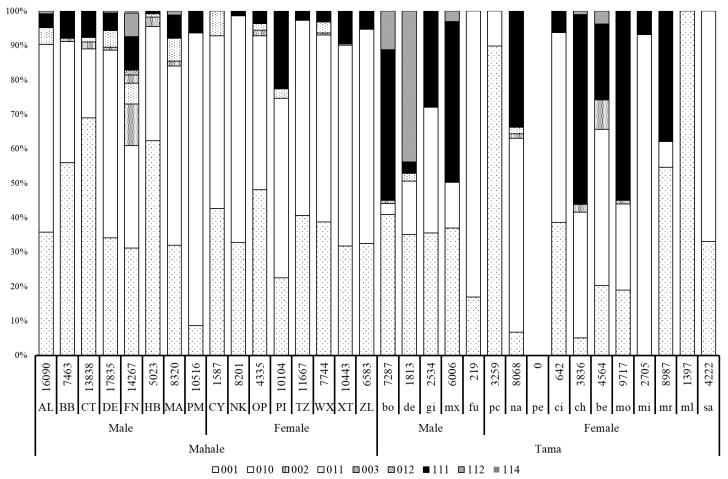
Percentage of time spent in each type of grooming clique for each individual in both groups. The numbers above the abbreviations of the focal individuals’ names indicate the total number of seconds that each individual participated in a social grooming interaction.

The list of all the observed grooming cliques is shown in Table 2, and the ratio of each grooming clique to social grooming for each focal individual is shown in Figure 2. In the observed types of grooming cliques, (111), (112), and (114) were mutual grooming, while the other types were one-directional grooming.

Since the total observation seconds of social grooming was extremely short, data of pe (0) and fu (219) in Tama were excluded from the statistical analysis. The mutual grooming ratio for each focal individual in Tama (26.8 ± 22.0%) was found to be significantly higher than those in Mahale (6.7 ± 5.7%) (Mann–Whitney’s *U* test, U = 59.5, df = 1, *p* = 0.030, Figure 2).

All the focal individuals in Mahale performed GHC at least once, while no individual did so in Tama during the study period.

### 3.3. Social Networks and Network Indices

**Figure 3:**
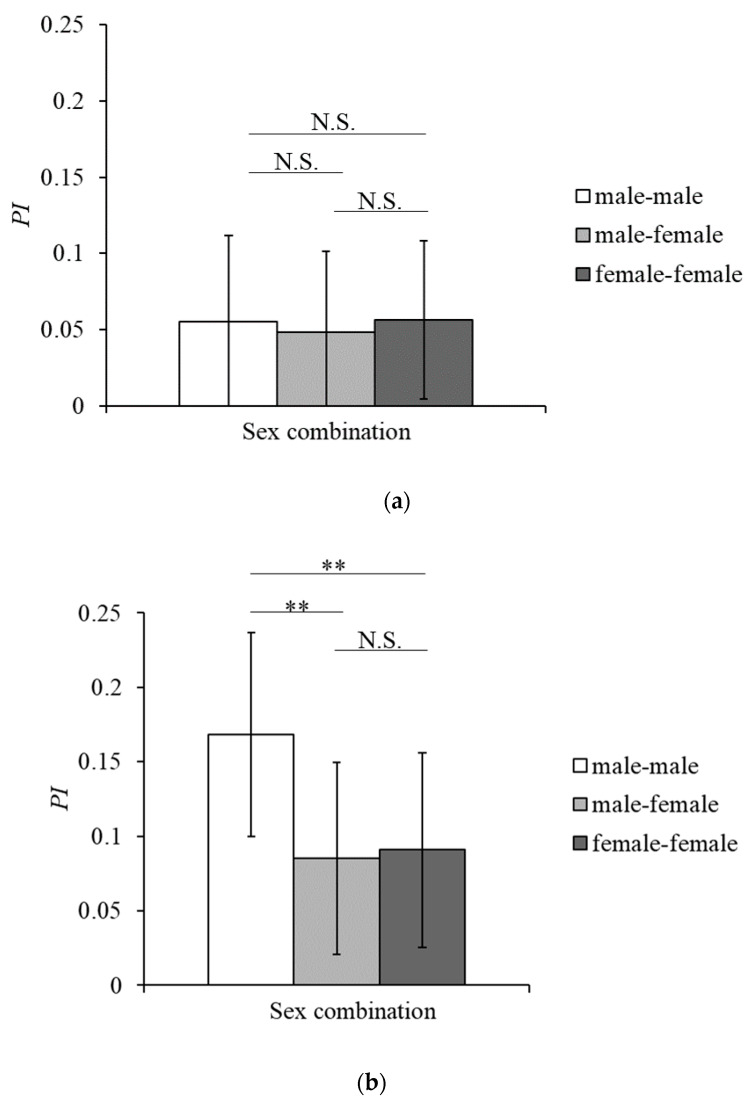
Proximity indexes (*PI*s) of each sex combination of focal individuals in Tama (**a**) and Mahale (**b**). Error bars indicate standard deviation. ** denotes a significance of *p* < 0.01, and N.S. denotes a nonsignificant (*p* > 0.05).

The *PI* matrices of the chimpanzees in Tama and Mahale are shown in Tables S2 and S3, respectively. In Tama, the *PI*s of each pair of focal individuals were not significantly different among their sex combinations (Kruskal–Wallis test, *χ*^2^ = 1.832, df = 2, *p* = 0.400, Figure 3a). In contrast, in Mahale, the *PI*s were significantly different among the sex combinations (Kruskal–Wallis test, *χ*^2^ = 35.947, df = 2, *p* < 0.001, Figure 3b). Multiple comparison between sex combinations by Holm’s method revealed that male–male *PI*s (0.168 ± 0.068) were significantly higher than the female–female *PI*s (0.091 ± 0.065) (*Z* = 4.276, *p* < 0.001) and male–female *PI*s (0.085 ± 0.065) (*Z* = 5.916, *p* < 0.001), whereas there were sno significant difference between female–female *PI*s and male–female *PI*s (*Z* = −0.873, *p* = 0.383).

**Figure 4:**
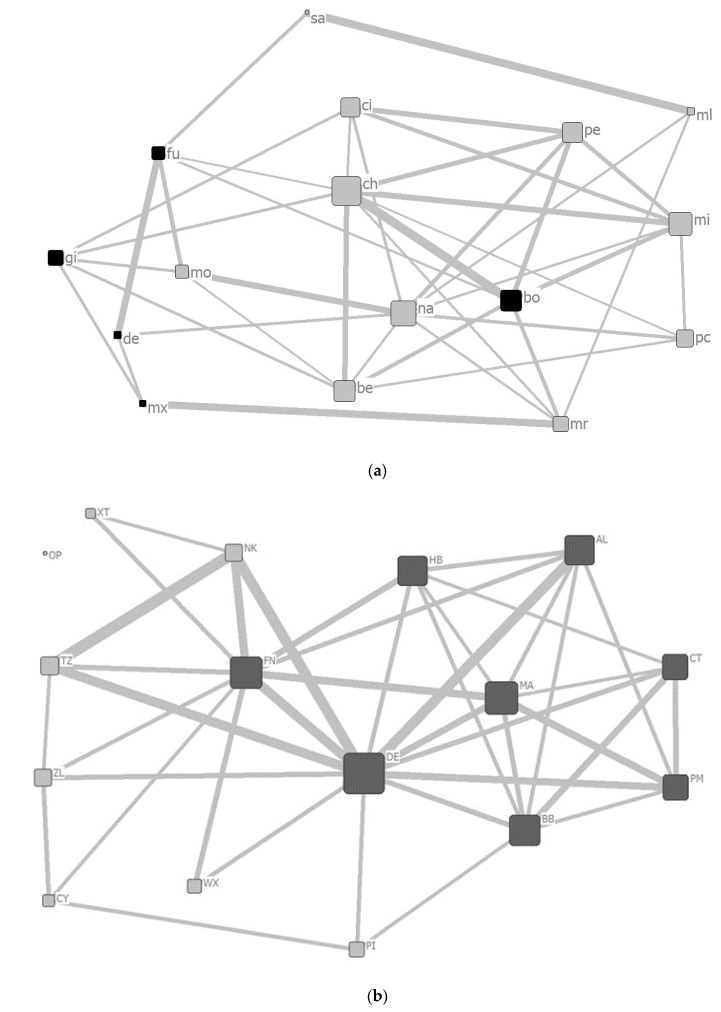
Social network based on the proximity between focal individuals in Tama (**a**) and Mahale (**b**). Black and gray squares represent males and females, respectively. The size of the square represents the value of the eigenvector centrality of each focal individual in the network. The thickness of the line is an indicator of the proximity index (*PI*) value between connected individuals. The abbreviation of each individual’s name is placed next to each square.

Using the *PI* matrices, social networks based on proximity for both groups are described in Figure 4. Using these networks, the eigenvector centralities for each focal individual of each group were calculated.

All of the focal individuals in Tama connected to at least one other individual in the social network and constituted one large cluster that contained all of the individuals, even when filtered, so that only *PI*s with values above the third quartile remained (Figure 4a). In Mahale, an adult female (OP) was depicted as isolated from the social network among the 16 focal individuals (Figure 4b). However, the *PI*s between OP and each of the other 15 individuals were greater than 0 (Table S3), indicating that OP and the other individuals had the opportunity to be in close proximity, albeit less frequently, during the study period.

The eigenvector centrality for each focal individual of males (0.325 ± 0.055) was found to be significantly higher than those of females (0.116 ± 0.053), in Mahale (randomization test, observed *t* = −7.2076, *p* < 0.001). However, the eigenvector centrality for each focal individual of males (0.182 ± 0.075) and females (0.251 ± 0.065), in Tama, were not significantly different (randomization test, observed *t* = 1.4101, *p* = 0.9057).

## 4. Discussion

### 4.1. Activity Budgets

As predicted, the activity budgets of collecting foraging of each individual in Tama were significantly lower than those in Mahale. There were no significant differences between the combined collecting, extractive, and hunting foraging percentages of the two groups, although hunting foraging was never observed in Tama.

Although the activity budgets for collecting foraging of captive chimpanzees tend to be shorter than those of wild individuals, when they rely only on scheduled baits that do not require processing [3,5,49], our results suggest that the multiple artificial feeders in Tama could have contributed to the increased duration of extractive foraging. Therefore, the total activity budget of foraging in captivity was approximately similar to that in the wild. Providing enrichment items to captive animals is considered to contribute to an increase in the time they spend eating and to an improvement in their physical, occupational, and sensory well-being [10]. The result that we found no abnormal behavior in Tama and Mahale suggests that the introduction of abundant and complicated enrichment items associated with foraging improved the well-being of captive chimpanzees and made abnormal behavior among them less likely to occur [9,39,40,50].

As predicted, the activity budgets of traveling for each individual in Tama were significantly lower than those in Mahale. One of the reasons why captive chimpanzees spent less time traveling than wild chimpanzees is that their outside enclosure was physically narrower than the home range of the wild groups. The ring area of M group chimpanzees, in Mahale, was 2700 ha, which is much larger than the 0.23 ha outside enclosure in Tama [19,36].

In contrast, previous studies have shown that there are large regional differences in the activity budgets for traveling in wild chimpanzees, from 6% in Budongo to 22% in the Tai Forest [51,52,53], depending on socio-ecological factors, such as individual density or food availability [54]. The values of activity budgets for traveling in Tama (12.0 ± 3.6%) and Mahale (15.4 ± 2.9%) in this study were within this range. Thus, the results suggest that the proportion of traveling in the daily activities of Tama chimpanzees was not necessarily lower than that of the wild chimpanzees in total. The area of outside enclosure is much larger than 0.15 ha, which is the international standard for 19 captive chimpanzees set by AZA (the Association of Zoos & Aquariums) [55]. Three-dimensional structures, such as towers that are 15 m high and ropes between them in the outside enclosure, on the one hand, allow the captive chimpanzees to generate behavioral diversity similar to that of wild chimpanzees spending a long time in the trees. On the other hand, however, it should be noted if excessive traveling is observed in chimpanzees living in enclosure, it can be a sign of stereotyping or stress.

### 4.2. Features of Grooming Interactions in Tama and Mahale

Contrary to what was predicted, the proportion of mutual grooming in the total social grooming of chimpanzees in Tama (26.8 ± 22.0%) was higher than that in Mahale (6.7 ± 5.7%). Comparable data are limited, based on our knowledge; only data on chimpanzees in Mahale studied from 1996 to 1997 are available [23], as well as data from Wamba for bonobos [56]. According to these previous studies, the mutual grooming ratio in Mahale chimpanzees in the past was 10.4% of total social grooming (recalculated from Figure 2 in Nakamura [22]), while that of wild bonobos in Wamba was approximately 0.27% (recalculated from Figure 2 in Sakamaki [56]). A comparison of our result with with these data, shows that our result of the mutual grooming ratio in Tama was suggested to be much higher than those of wild chimpanzees in Mahale, both in the present and the past, and accentuates the extremely low values in bonobos. Although a fission–fusion manner of variation in group membership is a common feature of the genus *Pan* [38,57], wild bonobos form a larger party with more stable memberships than those of wild chimpanzees [57,58]. Considering that most of the individuals in Tama live in the same outside enclosure during the day, our results were opposite those predicted by the hypothesis, i.e., that the uncertainty to encounter each other in the future would lead to a higher mutual grooming ratio [24,59].

One alternative explanation for the high mutual grooming ratio between individuals in Tama and the low ratio in Wamba is that the ratio could be an indicator of the degree of affiliative relationships between them [31,59,60]. Mutual grooming is thought to form a stronger social bond as it requires both partners to be actively involved and ensures reciprocity with respect to the amount of grooming between partners as opposed to one-directional grooming [22,24,60]. In addition, the number of affiliative interactions, such as mutual grooming, in captive chimpanzees, is likely be higher than in wild chimpanzees owing to crowding and the abundance of time that can be spent on social interaction in the more restricted area [8]. Future research is necessary to verify this possibility.

Our results suggest that social grooming idiosyncrasies could be present in Tama that have not been discovered yet due to focusing on GHC. Although mutual grooming is an interactive pattern that is a prerequisite for GHC, the results of this study suggest that groups with a high mutual grooming ratio do not necessarily exhibit GHC. Thus, the high mutual grooming ratio that occurs without GHC can be considered to be an idiosyncratic grooming variation in the groups in Tama [61,62,63].

### 4.3. Social Characteristics of Captive Chimpanzees

With the exception of OP in Mahale, each individual had at least two individuals in strong proximity to each other, and they formed one large cluster in both groups (Figure 3). These results suggest that both groups formed well-connected gathering networks [22,35].

**Figure 5:**
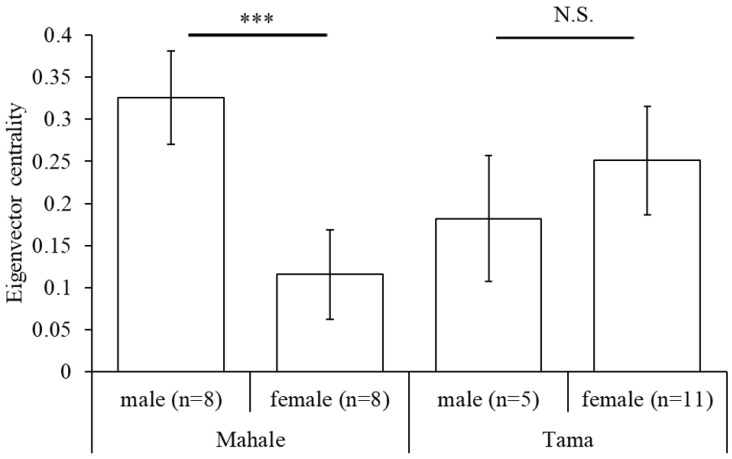
Comparisons of eigenvector centrality between males and females in each group. Error bars indicate standard deviation. *** denotes a significance of *p* < 0.001, and N.S. denotes a nonsignificant (*p* > 0.05).

The *PI*s of each pair of focal individuals in Tama were independent of the sex combination of the pairs (Figure 3a), while in Mahale, the male–male *PI*s were higher than the female–female and male–female *PI*s (Figure 3b). These results support the predictions about differences in proximity because of sex combinations between individuals. In addition, the eigenvector centralities of the social networks based on the proximity for males in Mahale were higher than those for females in Mahale, but no sex-specific differences were found in Tama (Figure 5). These results support the predictions about differences in position and core formation in the social network due to the sex of individuals.

The results from the Mahale chimpanzees, in this study, suggest that the males form the core of the multimale–multifemale group (Figure 4b), which is a finding that has often been reported in previous studies of chimpanzee societies in the wild [25,45,64]. In contrast, male chimpanzees in captivity do not need to cooperate, form a strong alliance with each other, or be aggressive toward other individuals in the group for a higher status [32,33,34]. Most of the members in Tama were always in close proximity to each other in their outside enclosures [65]. However, some of the combinations of individuals, such as bo and de, mo and pc, or fu and pc, were not kept in the outside enclosure at the same time to eliminate the possibility of accidents such as lethal struggles. Therefore, the *PI*s between them were all 0 (Table S2).

The differences in social relationships or social networks in Tama from those in Mahale could be due to the lack of hostile external groups and fierce conflicts with group members [29,30,66], as well as the need for alliances or the artificial migration system of individuals in zoos, which were different from those in the wild [1,3,32].

## 5. Conclusions

In Tama, chimpanzees attempted extractive foraging for food resources from multiple feeders, which suggests that the proportion of time spent foraging in total was closer to that of the wild chimpanzees. Bringing the proportion of time spent foraging closer to that of the wild chimpanzees is thought to inhibit the occurrence of abnormal behaviors [67], which was not observed in Tama. In addition, the proportion of time spent traveling in Tama was also in the range of those of wild groups, due to the three-dimensional structures or ropes between them in the outside enclosure, despite their narrow ranging area.

Environmental enrichment can help to simulate environmental conditions seen in the wild which can make the behavior of captive chimpanzees resemble that of wild groups. Thus, it is important to assess environmental enrichment for captive chimpanzees in terms of whether their behavior resembles that of wild groups, such as a high proportion of time spent foraging or traveling with no abnormal behaviors. From this perspective, the environmental enrichment for chimpanzees in Tama can be considered to have been successful [1,68,69]. The observed different social structures could be due to the differences in the living conditions in which the two groups live. In addition, the behavior of captive chimpanzees is generally limited by the available space and influenced by zookeepers, and thus, tends to have limited options for behavioral decision making and social partners. However, the idiosyncratic social grooming interactions found in Tama suggest that chimpanzees reared in a restricted and potentially stressful environment can use mutual grooming to build and maintain social cohesion and affiliative relationships [27,70].

## Figures and Tables

**Table 1:** The list of focal individuals in both study groups. * Tama, Tama Zoological Park, Tokyo; Wild, born in wild; Izu, Izu Shaboten Zoo, Shizuoka; KS, Kumamoto Sanctuary, Kumamoto; Noichi, Noichi Zoological Park, Kochi; M, M group in Mahale [36], K, K group in Mahale [36], O, a group other than M or K group [36].

Group	Name	Abbreviation	Sex	Age	Age Category	Place of Birth *
Tama	Bonbon	bo	Male	14	Young	Tama
	Deckie	de		app. 41	Adult	Wild
	Gin	gi		11	Young	Tama
	Max	mx		12	Young	Tama
	Fubuki	fu		5	Juvenile	Tama
	Peach	pc	Female	29	Adult	Izu
	Nana	na		36	Adult	Tama
	Peco	pe		app. 58	Adult	Wild
	Chico	ci		25	Adult	Tama
	Cherry	ch		29	Adult	Tama
	Berry	be		19	Adult	Tama
	Momoko	mo		26	Adult	KS
	Mikan	mi		13	Adult	Tama
	Marina	mr		29	Adult	KS
	Mil	ml		16	Adult	Tama
	Sakura	sa		10	Young	Noichi
Mahale	Alofu	AL	Male	20	Adult	M
	Bonobo	BB		21	Adult	M
	Carter	CT		17	Adult	M
	Kalunde	DE		39	Adult	M
	Fanana	FN		24	Adult	M
	Hanby	HB		22	Adult	M
	Masudi	MA		25	Adult	K
	Pimu	PM		14	Young	M
	Cynthia	CY	Female	20	Adult	O
	Nkombo	NK		32	Adult	K
	Opal	OP		31	Adult	O
	Pinky	PI		30	Adult	O
	Totzy	TZ		20	Adult	M
	Wakusi	WX		41	Adult	O
	Christina	XT		27	Adult	O
	Zola	ZL		15	Adult	O

**Table 2:** Types of grooming cliques during the study period. Black circles indicate the focal individual, white circles indicate the other individuals, → indicates who the grooming was directed toward, ↔ indicates mutual grooming, ○○ or ○○○ indicates two or three individuals simultaneously grooming a focal individual. The gray rows indicate mutual grooming.

Code of Clique Types	Configuration of Participating Individuals	Number of Participating Individuals
001	○→●	2
010	●→○	2
111	●↔○	2
011	○→●→○	3
002	○○→●	3
112	○→●↔○	3
003	○○○→●	4
012	○○→●→○	4
114	○○○→●↔○	5

## Supplementary Materials

The following are available online at https://www.mdpi.com/2076-2615/10/6/1063/s1, Table S1: Summary of the total number of total observation days and the total observation hours for each time period of each focal individual for each group. Table S2: Proximity index (PI) matrix for all focal individuals in Tama. The first quartile, mean, and third quartile were 0.0168, 0.0525, and 0.0653, respectively. Table S3: Proximity index (PI) matrix for all focal individuals in Mahale. The first quartile, mean, and third quartile were 0.0560, 0.0745, and 0.1296, respectively.
